# Hypertriglyceridemia-Induced Acute Pancreatitis Exacerbated by Combined Oral Contraceptive Use: A Case Report

**DOI:** 10.7759/cureus.64537

**Published:** 2024-07-14

**Authors:** Shreya Bhatt, Aleymi Perez, Evan Sarmiento, Taylor Alfonso, Sahil Shah, Sergio Hernandez Borges

**Affiliations:** 1 Osteopathic Medicine, Nova Southeastern University Dr. Kiran C. Patel College of Osteopathic Medicine, Fort Lauderdale, USA; 2 Osteopathic Medicine, Lake Erie College of Osteopathic Medicine, Bradenton, USA; 3 Pain Management & Rehabilitation, Larkin Community Hospital Palm Springs Campus, Miami, USA; 4 Internal Medicine/Family Medicine, Larkin Community Hospital Palm Springs Campus, Miami, USA

**Keywords:** familial hypertriglyceridemia, hypertriglyceridemic pancreatitis, hypertriglyceridemia (htg), combined oral contraceptives, acute pancreatitis

## Abstract

Acute pancreatitis can be induced by a vast variety of etiologies including its more common causes such as cholelithiasis and alcohol abuse, but in certain cases it can also be secondary to hypertriglyceridemia. Additionally, combined oral contraceptive use can enhance the severity of hypertriglyceridemia-induced acute pancreatitis (HTG-AP). The data between this association is much more limited than the more common causes of acute pancreatitis. In this case, we aim to highlight the onset of hypertriglyceridemia-induced acute pancreatitis due to recent combined oral contraceptive use in a 34-year-old Hispanic female patient with a family history of hypertriglyceridemia. With the initiation of a low-fat diet, insulin regimen, and lipid-lowering medications, she was able to significantly improve her elevated triglyceride levels from 3772 to 440 throughout the duration of her six-day hospital stay. Due to the less commonly known relationship between combined oral contraceptive use and HTG-AP, this case serves to enhance understanding of the pathophysiology of this condition, the appropriate diagnostic evaluation, and the associated treatment options to optimize patient care and create efficacious management plans. By increasing awareness of this association, patients with familial hypertriglyceridemia can be made aware of the risks of combined oral contraceptive use to accordingly prevent complications and improve clinical outcomes.

## Introduction

Acute pancreatitis is an acute inflammatory process characterized by severe abdominal pain, nausea, vomiting, and elevated pancreatic enzymes. The most common causes of acute pancreatitis are alcohol abuse and cholelithiasis. However, in this case study we present a patient with a family history of hypertriglyceridemia who came to the emergency department with a hypertriglyceridemia-induced acute pancreatitis (HTG-AP) exacerbated by combined oral contraceptive use. Hypertriglyceridemia is defined as fasting serum triglyceride levels above 160 milligrams per deciliter (mg/dL). The risk of acute pancreatitis due to hypertriglyceridemia increases to approximately 5% with levels above 1000 mg/dL and rises 10-20% with levels greater than 2000 mg/dL [1].

The etiology of hypertriglyceridemia can be divided into two categories: primary and secondary. Primary HTG includes the genetic susceptibility to elevated triglycerides from familial chylomicronemia syndrome, primary hypertriglyceridemia, and mixed hypertriglyceridemia also known as Fredrickson Type I, IV, and V, respectively [2,3]. Fredrickson’s classification of the abnormal lipoprotein patterns have been designated as types I-V to correlate the phenotypes and clinical manifestations to the appropriate genotype. A Fredrickson Type I diagnosis is made by the presence of elevated chylomicronemia on a normal fat intake, classified as 35 to 45% of total daily calories, which is usually detected in childhood. These individuals have low activity of lipoprotein lipase in their adipose tissue leading to a decrease in the clearance of triglycerides. Fredrickson Type IV involves a familial increase in pre-beta lipoprotein and a decreased ability to clear pre-beta lipoproteins from the plasma which leads to a hypertriglyceridemia when the patient’s fat intake is high, classified as greater than 45% of total daily calories. Fredrickson Type V is characterized by a defect in metabolism of both exogenous and endogenous triglycerides for which there is an excess of both chylomicrons and pre-beta lipoproteins when the patient has a normal fat intake [4]. Secondary factors include alcohol abuse, obesity, uncontrolled diabetes, hypothyroidism, and drugs like estrogen as seen in our patient [2,3]. Estrogen can at times lead to severe acute pancreatitis through two mechanisms. First, it increases blood triglyceride levels by augmenting very low-density lipoprotein secretion and reducing hepatic triglyceride lipase. Secondly, estrogen causes a hypercoagulable state inducing pancreatic necrosis [5,6].

The pathophysiology of hypertriglyceridemia-induced pancreatitis remains poorly understood but is believed to be due to the metabolism of excessive triglycerides by pancreatic lipase to free fatty acids leading to pancreatic cell injury and ischemia [2]. In this case study, we include the clinical presentation, diagnostic evaluation, management strategies, and outcomes for a patient with HTG-AP. Through an in-depth exploration of this case, we aim to enhance the understanding of HTG-AP and demonstrate a predicament in which other factors aside from alcohol abuse or cholelithiasis can lead to a serious case of acute pancreatitis.

## Case presentation

A 34-year-old Hispanic female, gravida one, para one, with a past medical history significant for hypertriglyceridemia presented to the emergency department complaining of worsening abdominal pain that began two days prior in the epigastric region. The night before she reported having chills and since the onset of her pain she was having nausea and vomiting. She does not take any home medications aside from combined oral contraceptive pills that were started three months prior to her hospitalization. At the time when her triglyceride counts were elevated two years before, she could not continue treatment because she became pregnant. She also has a family history of hypertriglyceridemia in her grandmother.

**Figure 1:**
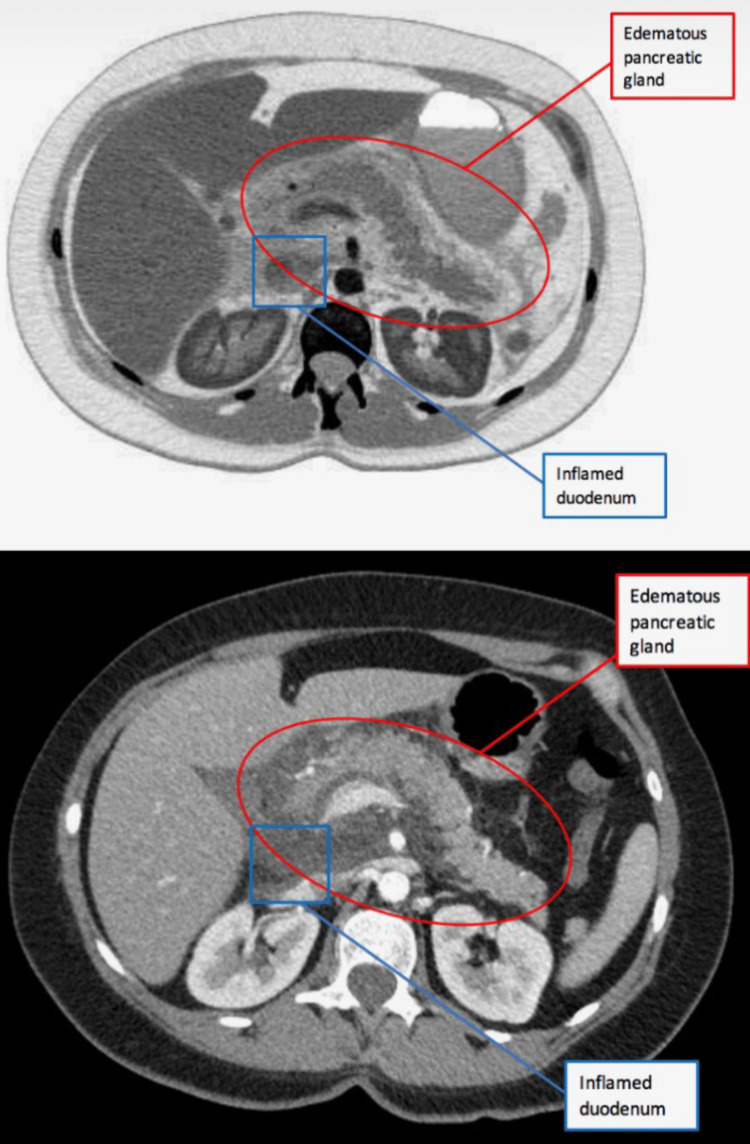
CT Scan of Abdomen in the transverse plane showed findings consistent with uncomplicated pancreatitis and inflammatory changes in the duodenum most likely as a secondary reaction to the adjacent acute pancreatitis

**Figure 2:**
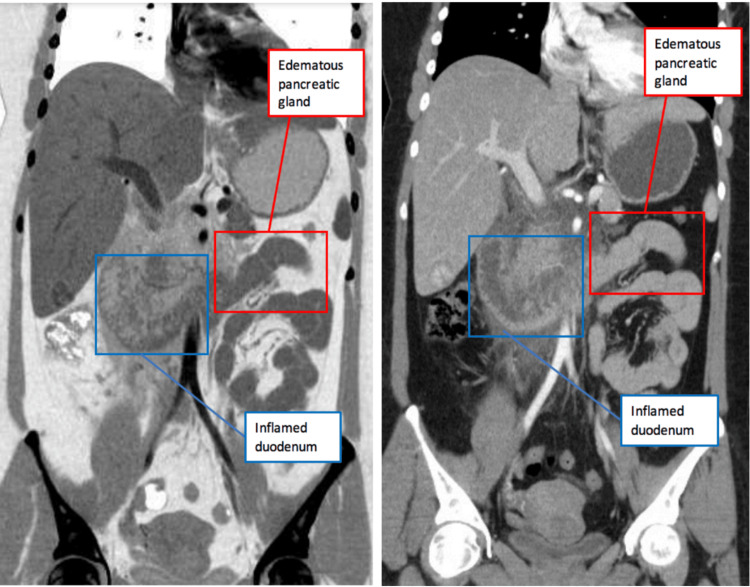
CT Scan of Abdomen in the coronal plane showed findings consistent with uncomplicated pancreatitis and inflammatory changes in the duodenum most likely as a secondary reaction to the adjacent acute pancreatitis

**Figure 3:**
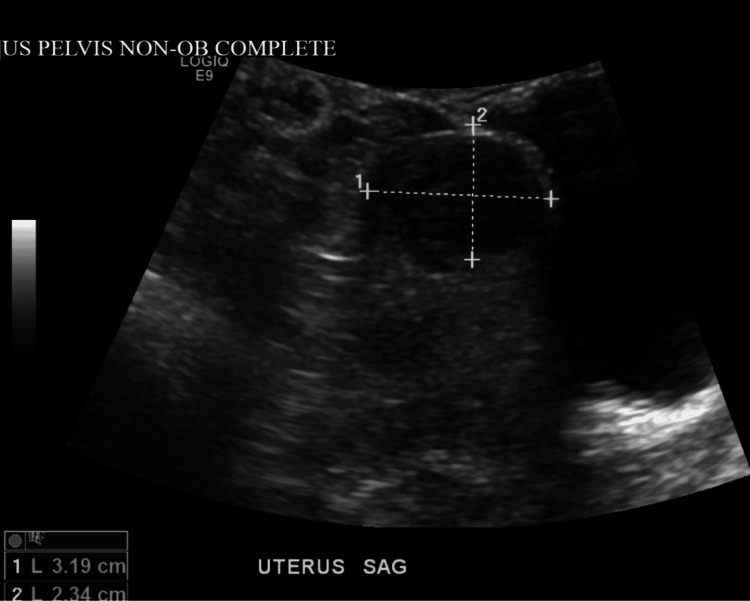
Pelvic ultrasound showing a 3.2 centimeter intramural fibroid at the uterine fundus

Upon arrival to the emergency department her vital signs showed a blood pressure of 132/84 mmHg, heart rate of 120 beats per minute, temperature of 99.8 degrees Fahrenheit, and an oxygen saturation of 95% on room air. Her labs showed an elevated lipase of 1142 and her triglyceride count on admission was at a value of 3772 (see Table 1 for comparison of the patient’s lab values at admission and discharge with respect to normal lab values). Her complete blood count revealed an elevated leukocyte count of 18.9, neutrophil percentage of 82.5%, mean corpuscular hemoglobin concentration (MCHC) of 37.6, and hemoglobin of 15.6. Her erythrocyte count of 4.47, platelet count of 259, mean red blood cell volume (MCV) of 92.8, mean hemoglobin amount per red blood cell (MCH) of 34.9, and hematocrit (HCT) of 41.5 all were within normal limits. Her lymphocyte percentage was low at 11.8%. She was tested for coronavirus disease 2019 (COVID-19) and the results came back negative. Her electrolytes showed a sodium level of 136, potassium of 4.3, chloride of 104, and calcium of 8.9 which were all within normal limits. Her glucose was slightly elevated at a level of 130. Her amylase levels were not collected, but her liver function tests revealed an albumin of 4.1, alkaline phosphatase of 95, alanine transaminase of 17, and aspartate transaminase of 20. Blood cultures were ordered and came back negative. Over the duration of her six-day hospital stay, her triglyceride count trended down as shown in Table 1. On imaging, her CT scan of the abdomen showed findings consistent with uncomplicated acute pancreatitis and inflammatory changes in the duodenum most likely as a secondary reaction to the adjacent pancreatitis (see Figure 1 and Figure 2). She had bibasilar-dependent opacities that favored atelectasis, and aspiration pneumonia was considered due to her episodes of vomiting. There was also an incidental finding of a 1.8 centimeter (cm) liver lesion consistent with benign focal nodular hyperplasia. An ultrasound of the pelvis showed a 3.2 cm intramural fibroid at the uterine fundus (see Figure 3).

**Table 1:** Pertinent Lab Values MCHC: mean corpuscular hemoglobin concentration, MCV: mean red blood cell volume, MCH: mean hemoglobin amount per red blood cell

Laboratory Investigation	Patient Result on Admission	Patient Result at Discharge	Reference Range [7]
Lipase (units/liter)	1142	131	<95
Triglyceride Count Trend (milligram/deciliter) [over 6 days, with two measurements taken on Days 3 and 4]	3772 → 2081 → 1343 → 984 → 895 → 759 → 649 → 440		<150
Leukocyte Count (per microliter)	18,900	7,800	4000-10000
Neutrophil Percentage (%)	82.5	60.3	40-60
MCHC (grams/deciliter)	37.6	32.1	32-36
Hemoglobin (grams/deciliter)	15.6	10.4	12-16
Erythrocyte Count (per microliter)	4.47	3.38	4.2-5.9
Platelet Count (per liter)	259	278	150-350
MCV (fluid ounce)	92.8	95.9	80-100
MCH (picogram)	34.9	30.8	28-32
Hematocrit Percentage (%)	41.5	32.4	36-47
Lymphocyte Percentage (%)	11.8	23.8	20-40
Sodium (milliequivalent/liter)	136	137	136-145
Potassium (milliequivalent/liter)	4.3	3.6	3.5-5.0
Chloride (milliequivalent/liter)	104	108	98-106
Calcium (milligram/deciliter)	8.9	8.2	9-10.5
Glucose (milligram/deciliter)	130	111	70-100
Albumin (grams/deciliter)	4.1	3.1	3.5-5.5
Alkaline Phosphatase (units/liter)	95	95	36-92
Alanine Transaminase (units/liter)	17	39	0-35
Aspartate Transaminase (units/liter)	20	46	0-35

During her stay she was transferred to the Intensive Care Unit and given dextrose 10% and dextrose in lactated Ringer’s 5%, hydromorphone 4 milligrams (mg) intravenously every six hours as needed for pain management, ceftriaxone 1 gram daily due to Klebsiella pneumonia findings in her urine culture, and was placed on an insulin drip to further manage her hypertriglyceridemia as insulin activates lipoprotein lipase and helps break down the triglycerides. On her second day at the hospital, she had a nothing by mouth (NPO) order that lasted two days. After this period, she was started on a low-fat diet, daily fenofibrate 200 mg, atorvastatin 20 mg at night daily, and omega-3-acid ethyl esters 2 grams twice a day to lower her lipid levels. 

Due to the intricacies of her diagnosis, the endocrinology, cardiology, pulmonology/critical care, gastroenterology, and general surgery teams were consulted. The endocrinology team agreed with the plan set forth by the internal medicine team to initially start the patient on IV fluids and an insulin drip. They also discussed that the patient should use alternative contraceptive methods such as an intrauterine device and encouraged her to have a nutritionist evaluation prior to discharge to ensure that she understood the importance of being on a very low-fat diet along with moderate intensity exercise 30 minutes a day for five days every week. The cardiology team was consulted due to persistent tachycardia ranging from 120 to 140 beats per minute. A comprehensive 2D, Doppler, and color-flow echocardiogram was ordered revealing no anatomical abnormalities, no signs of pleural effusion or endocarditis, and an estimated left ventricular ejection fraction of 55%. The etiology of her tachycardia was likely due to an underlying urinary tract infection and dehydration. She was prescribed metoprolol 50mg twice daily for her rate control. Since she needed an insulin drip, she was transferred to the Intensive Care Unit where the pulmonology and critical care teams detected an elevated D-dimer of 13.2 on the second night of her stay. However, after conducting a CT Angiogram of the chest, no evidence of an acute pulmonary embolism, aortic aneurysm, or aortic dissection was found. The gastroenterology team was consulted due to the patient’s diagnosis of uncomplicated acute pancreatitis secondary to hypertriglyceridemia. They agreed that the patient should receive supportive care with fluid resuscitation and pain control until she could tolerate an oral diet after which her dietary fat should be severely restricted at a level of <5% fat until her triglyceride levels were below 1000 mg/dL. The general surgery team was consulted, but found that no urgent surgical intervention was required. All the consulted specialties monitored the patient for any changes and ensured that she received the adequate measures necessary to ameliorate any pain and discomfort she was experiencing.

Over the span of her six days at the hospital, she had significant improvements in her condition and reported feeling a lot better with no complaints of abdominal pain, nausea, vomiting, melena, fever, or chills. Her labs prior to discharge showed elevated levels of low-density lipoprotein (LDL) at 137, chloride of 108, lipase of 131, triglycerides of 440, alkaline phosphatase of 95, alanine transaminase of 39, aspartate transaminase of 46, and glucose of 111, low levels of calcium at 8.2, blood urea nitrogen of 3, albumin of 3.1, erythrocyte count of 3.38, hematocrit of 32.4, and hemoglobin of 10.4, and normal levels of sodium at 137, potassium of 3.6, and creatinine of 0.53. Although all of her lab values were not within normal limits yet, she was in no acute distress. She requested to be discharged so she can be home with her baby and continue the rest of her care at an outpatient clinic. At discharge, she was sent home with atorvastatin 20 mg by mouth at bedtime, oral ciprofloxacin 500 mg for her Klebsiella pneumonia, fenofibrate 145 mg by mouth daily, and metoprolol 20 mg by mouth twice daily. She was recommended to continue her lipid-lowering medications, a low-fat diet, and to avoid oral estrogen to prevent recurrence of her acute HTG-AP. She was advised to obtain an MRI of the abdomen in an outpatient clinic to further evaluate the incidental liver lesion that was highly suggestive of focal nodular hyperplasia as well as an outpatient visit to the gastroenterologist. Due to her young age, recent oral contraceptive use, and medical and family history of hypertriglyceridemia, further evaluation to understand the etiology of her condition was pursued.

## Discussion

Hypertriglyceridemia is the third most common cause of acute pancreatitis following cholelithiasis and alcohol consumption. When triglyceride levels are >1000 mg/dL, it is categorized as severe hypertriglyceridemia [8]. It has been reported that patients with severe acute pancreatitis experience hypertriglyceridemia and elevated D-dimers secondary to elevated triglycerides and inflammatory reactions [9]. In the case described above, the patient presented to the emergency department with acute pancreatitis caused by triglyceride levels >3700 mg/dL. The severity was further indicated by an elevated D-dimer of 13.2 on the second night of her visit.

In this case, the patient presented an interplay of both primary and secondary risk factors that contributed to her acute pancreatitis. The patient’s family history of hypertriglyceridemia and her past medical history of elevated triglycerides indicated a high likelihood of having familial hypertriglyceridemia, which can be further explored through a hypertriglyceridemia gene panel to look at pathogenic variants involved in triglyceride metabolism [10]. Familial hypertriglyceridemia is inherited in an autosomal dominant fashion with variable penetrance, leading to a deficit in the breakdown of very low-density lipoproteins (VLDLs), causing elevated triglycerides with large VLDL particle size [11]. Although she was previously on lipid-lowering medication, the discontinuation of those medications during pregnancy and in the postpartum period led to a lack of effectiveness in controlling her triglyceride levels. 

There are medications such as combined oral contraceptives that have been associated with elevated triglyceride levels due to their estrogen component [6,12]. Estrogen raises serum triglyceride levels by lowering hepatic triglyceride lipase, and promoting hepatic production of triglycerides which are released into the circulation through VLDLs [6]. Acute pancreatitis can manifest within a week of initiating such medications, however, some agents may take as long as weeks to even months to trigger acute pancreatitis [8]. Her initiation of oral contraceptives three months prior posed a risk factor for developing HTG-AP. For this reason, her combined oral contraceptive was discontinued and the endocrinology team at the hospital advised this patient of alternative birth control options. They also recommended that she never take medications that include oral estrogen again.

Management of acute pancreatitis begins with conservative treatments. This includes monitoring oxygen saturation with a goal of 94-98% saturation. Bowel rest with no oral intake accelerates the clearance of elevated triglycerides and reduces pancreatic excretions. Administration of intravenous fluids functions to correct third space volume loss and tissue hypoperfusion. Furthermore, analgesics such as opioids are appropriate for pain control [2,12,13].

Due to hypertriglyceridemia being the cause of this patient’s acute pancreatitis, the initial treatment goal was to lower triglyceride levels. Administration of intravenous insulin lowers triglycerides by accelerating chylomicron degradation via its activation of lipoprotein lipase activity. This reverses the stress-associated lipotoxicity caused by the breakdown of triglycerides into free fatty acids [1]. Additionally, the rapid discontinuation of the offending agent, such as this patient’s oral contraceptives, decreases complications and shortens the length of hospital stay [6]. Although not used in this case, heparin and plasmapheresis can be an additional part of the patient’s management to lower serum triglyceride levels [2].

The long-term management to prevent recurrence of acute pancreatitis involves various modalities of treatment. The primary goal is to maintain triglyceride levels below 500 mg/dL. Lipid-lowering agents are commonly used to decrease triglyceride levels. Specifically, fibrates, such as fenofibrate, show the greatest efficacy at lowering triglycerides, and therefore are first-line agents for treating severe hypertriglyceridemia [1,2]. Fibrates lower triglyceride and cholesterol levels primarily by binding to peroxisome proliferator-activated receptors (PPARs), leading to activation of transcription factors. This action results in various effects, including the induction of lipoprotein lipolysis, increased hepatic fatty acid reuptake, and reduced hepatic triglyceride production. Additionally, fibrates enhance the affinity of LDL to LDL receptors increasing the removal of circulating LDLs, while also increasing high-density lipoproteins (HDLs) by stimulating the liver to produce apoA-I and apoA-II. Furthermore, the reduction of plasma triglyceride levels may result from the decreased exchange between VLDL and HDL [14]. In addition to fibrates, other treatment options include niacin, omega-three fatty acids, and statins [1,2]. Lifestyle modifications must be enacted as well, such as starting a low-saturated-fat diet, weight loss, and avoidance of alcohol. Furthermore, secondary factors such as having the patient’s glucose levels under control, and avoiding medications that are associated with elevating triglyceride levels such as estrogen, diuretics, and beta adrenergic blockers, can be beneficial in avoiding HTG-AP episodes [1,12,13].

## Conclusions

This case shows how imperative it is for healthcare providers to recognize a patient’s risk factors for hypertriglyceridemia, requiring careful adjustment or implementation of medication regimens. Acquiring a genetic test that can identify pathologic variants, such as a hypertriglyceridemia gene panel, can help highlight the underlying cause of hypertriglyceridemia. It is important to educate patients on the consequences of discontinuing lipid-lowering medications, and the side effects of oral contraceptives on triglyceride levels. By addressing these factors proactively, providers can reduce the risks that come with hypertriglyceridemia such as acute pancreatitis and avoid other serious life-threatening complications.
